# Mutational spectrum in congenital dyserythropoietic anemia type II: Identification of 19 novel variants in *SEC23B* gene

**DOI:** 10.1002/ajh.21866

**Published:** 2010-12

**Authors:** Roberta Russo, Maria Rosaria Esposito, Roberta Asci, Antonella Gambale, Silverio Perrotta, Ugo Ramenghi, Gian Luca Forni, Vedat Uygun, Jean Delaunay, Achille Iolascon

**Affiliations:** 1 CEINGE Biotecnologie AvanzateNapoli, Italy; 2 Department of Biochemistry and Medical Biotechnologies, University Federico II of NaplesItaly; 3 Department of Pediatrics, Second University of NaplesItaly; 4 Hematology Unit, Department of Pediatrics, University of TorinoTorino, Italy; 5 Centro della Microcitemia e Anemie Congenite, Ospedale GallieraGenova, Italy; 6 Department of Pediatric Hematology-Oncology, Akdeniz University School of MedicineAntalya, Turkey; 7 INSERM U 779, Faculté de Médecine Paris-Sud, Univ Paris-SudLe Kremlin-Bicêtre, France

## Abstract

*SEC23B* gene encodes an essential component of the coat protein complex II (COPII)-coated vesicles. Mutations in this gene cause the vast majority the congenital dyserythropoietic anemia Type II (CDA II), a rare disorder resulting from impaired erythropoiesis. Here, we investigated 28 CDA II patients from 21 unrelated families enrolled in the CDA II International Registry. Overall, we found 19 novel variants [c.2270 A>C p.H757P; c.2149−2 A>G; c.1109+1 G>A; c.387(delG) p.L129LfsX26; c.1858 A>G p.M620V; c.1832 G>C p.R611P; c.1735 T>A p.Y579N; c.1254 T>G p.I418M; c.1015 C>T p.R339X; c.1603 C>T p.R535X; c.1654 C>T p.L552F; c.1307 C>T p.S436L; c.279+3 A>G; c. 2150(delC) p.A717VfsX7; c.1733 T>C p.L578P; c.1109+5 G>A; c.221+31 A>G; c.367 C>T p.R123X; c.1857_1859delCAT; p.I619del] in the homozygous or the compound heterozygous state. Homozygosity or compound heterozygosity for two nonsense mutations was never found. In four cases the sequencing analysis has failed to find two mutations. To discuss the putative functional consequences of missense mutations, computational analysis and sequence alignment were performed. Our data underscore the high allelic heterogeneity of CDA II, as the most of *SEC23B* variations are inherited as private mutations. In this mutation update, we also provided a tool to improve and facilitate the molecular diagnosis of CDA II by defining the frequency of mutations in each exon. Am. J. Hematol., 2010. © 2010 Wiley-Liss, Inc.

## Introduction

Mutations in the *SEC23B* gene (Sec23 homolog B; MIM# 610512, 20p11.23) cause the vast majority of the congenital dyserythropoietic anemia Type II (CDA II MIM# %224100), an autosomal recessive disorder that represents the most common form of CDAs. Like other forms, it is characterized by a mild to moderate lifelong anemia, ineffective erythropoiesis, and morphologic abnormalities of mature red blood cells (RBC) and their precursors [1]. Splenomegaly and jaundice are the most evident clinical signs [2,3]. About 10% of patients require frequent transfusions or are dependent on them. Until now, it has not been possible to establish whether these cases are due to more severe mutations or whether they are due to other gene(s) causing the same phenotype, or to the interaction with other intra-erythrocytic defects, such as thalassemia [4].

CDA II is associated with the presence in the bone marrow of bi- or multinucleated late erythroid precursors, karyorrhexis, and pseudo-Gaucher cells. Diagnosis was, for a long time, achieved by bone marrow examination. It shows 5–10 times more erythroblasts than normal (erythroid hyperplasia) [1,3]; early erythroblasts are relatively normal, but more than 10% of all erythroid cells are binucleated with equal size of two nuclei or multinucleated [5]. Extensive morphological anomalies of CDA-II erythroblastic cells were observed using electron microscopy (EM), with the most significant being the presence of the so-called double membrane, which is observable in mature RBCs as well [6,7]. This typical aspect is due to residual endoplasmic reticulum [8]. Polyacrylamide gel electrophoresis in presence of sodium dodecyl sulphate (SDS-PAGE) revealed the presence of a thinner band 3 with an increased anodic mobility due to reduced glycosylation. This cardinal abnormality represents a key for the diagnosis [9] and could suggest a defect in vesicles trafficking.

SEC23B is an essential component of coat protein complex II (COPII)-coated vesicles that transport secretory proteins from the endoplasmic reticulum (ER) to the Golgi complex. In *Saccharomyces cerevisiae* this complex is well fine characterized: the coat comprises five subunits, the small GTPase Sar1 (Secretion associated, ras-related), the Sec23–Sec24 “inner coat” complex, and the Sec13–Sec31 “outer coat” complex. Sec23 is a GTPase-activating protein (GAP) that activates Sar1 [10], and the outer coat is responsible for stimulating this GAP-activity. Therefore, full assembly of the coat stimulates Sar1 GTP-hydrolysis activity [11]. This enables the coat to depolymerise and be recycled for another round of vesicle biogenesis.

The *SEC23B* gene spans ∼54 kb on human chromosome 20p11.23 and is composed of 20 exon regions codifying 767 residues arranged in five functional domains: zinc finger, trunk, β-sheet, helical, and gelsolin domain. Each domain interacts with at least one of three coat subunits, in particular, trunk domain is linked by Sec24, Sar1, and Sec31, gelsolin domain is linked by Sar1 and Sec31, and other three domains are linked only by Sec31.

To date, 34 causative mutations in *SEC23B* gene were described [12–15]. In this work, we described 19 novel variants localized along the entire coding sequence (eight missense, two frameshift, three nonsense, five splicing mutations, and one small deletion). This is the first update of *SEC23B* mutations involved in CDA II. Moreover, we attempted to characterize the putative effects of the amino acid substitutions on protein structure or function, as well as on mRNA stability by using bioinformatic analyses; we also tried to define the effect of mutations near splice site on mRNA processing alteration by using the same approaches. Finally, by defining the frequency of mutations and those of mutated alleles in each exon, we provided a powerful tool to improve the molecular diagnosis of CDA II.

## Methods

### Inclusion criteria and definition of the cohort used for mutational analysis

Twenty-eight CDA II patients (15 males and 13 females) from 21 unrelated families enrolled in the CDA II International Registry were investigated (Supporting Information Table Is). The diagnosis of CDA II was based on the presence of mild to moderate anemia, ineffective erythropoiesis, and morphological abnormalities of the erythroblasts in the bone marrow. Confirmation of the diagnosis was made by mutation screening of the *SEC23B* gene or at least one of the following analyses: the revelation of the typical narrower band size and faster migration of the band 3 and band 4.5 proteins at SDS-PAGE; the demonstration of superficial appearance of reticulum-endothelial proteins (calreticulin, glucose regulated protein 78, protein disulphide isomerase) on membrane proteins by Western blot (WB) analysis; the presence of a discontinuous double membrane in mature erythroblasts by electron microscopy (EM) [5].

After signed informed consent, blood was obtained for genetic analysis from the probands. Blood from healthy control subjects was obtained after signed consent according to the Declaration of Helsinki. This project was approved by local ethical committee (University Federico II).

### Genomic mutational screening

Genomic DNA preparation, mutational search, oligonucleotide primers design, and direct sequencing were performed as previously described [14]. Sequence primers are available on request (achille.iolascon@unina.it). Nucleotide numbering reflects cDNA numbering with +1 corresponding to the A of ATG translation initiation codon in the reference sequence (Ensembl transcript ID: ENST00000377475). The initiation codon is codon 1.

### In silico analyses

To perform a multiple sequence alignment, sequences homologous to human SEC23B were assembled in a multiple sequence alignment using database UniProtKB release 15.0 of March 25, 2009; program NCBI BLASTP 2.0.12 on http://services.uniprot.org (http://services.uniprot.org/blast/). The alignment is made using sequences found in the Uniprot Knowledge-base. The aligned SEC23 sequences were from the species: human (Accession No. Q15437), mouse (Q9D662), *Xenopus laevis* (Q6DJE0), *Danio rerio* (Q6AZ98), *Caenorhabditis elegans* (Q9U2Z1), *Saccharomyces cerevisiae* (B3LKE0). The secondary structure of SEC23B protein was predicted by using Jpred, a web server that takes a protein sequence and from this predicts secondary structure using a neural network called Jnet (http://www.compbio.dundee.ac.uk/www-jpred/). To localize the missense and nonsense alterations within the protein domains of SEC23B, we submitted the wild type protein sequence on ESyPred3D Web Server 1.0 (http://www.fundp.ac.be/sciences/biologie/urbm/bioinfo/esypred/). This tool implements a homology modeling approach followed by a final analysis with MODELLER release 4 to build a 3D model of the submitted protein [16]. This routine includes the satisfaction of spatial and geometric restraints and a very fast dynamic annealing. The 3D structures were visualized using the molecular-graphics software Yasara (http://www.yasara.org).

The possible impact of the amino acid substitution on the structure or function of protein was predicted by using two bioinformatic tools specifically designed for interpretation of missense variants: PolyPhen (http://genetics.bwh.harvard.edu/pph/and) [17] and PANTHER (http://www.pantherdb.org/tools/csnpScoreForm.jsp). PolyPhen identifies homologues of the input sequence via BLAST search and computes the absolute value of the difference between profile scores of both allelic variants in the mutated position. Big values of this difference (PSIC score) may indicate that the studied substitution is rarely or never observed in the protein family. For the input form, we used the protein identifier of SEC23B (Q15437) from the UniProt database. PANTHER calculates the subPSEC (substitution position-specific evolutionary conservation) score based on an alignment of evolutionarily related proteins. This score is the negative logarithm of the probability ratio of the wild-type and mutant amino acids at a particular position. PANTHER subPSEC scores are continuous values from 0 (neutral) to about −10 (most likely to be deleterious). Data from PANTHER can be used to calculate the probability that a given variant will have a deleterious effect on protein function (*P*_deleterious_): a subPSEC score of −3 corresponds to a *P*_deleterious_ of 0.5 [18].

To analyze the possible effect of the mutations near to 3′ and 5′ splice site junctions on splicing processing, we used a web server tool, Splice Site Prediction by Neural Network (http://www.fruitfly.org/seq_tools/splice.html). The output of the network is a score between 0 and 1 for a potential splice site.

Secondary structures of the full-length SEC23B mRNA, either wild type sequence that those mutated, were predicted by the program GeneBee (http://www.genebee.msu.su/genebee.html).

### Frequency of the mutations assessment

The relative richness in mutations (i) has been calculated by the ratio of the mutations falling in each exon of *SEC23B* gene and the overall count of causative mutations identified until now; similarly, the frequency of mutated alleles in each exon (ii), by the ratio of the number of mutated alleles for each exon and the overall count of mutated alleles. For the mutations near to 3′ and 5′ splice site junctions, the exon flanking splice junction has been considered. The definition of “exon” includes about 200 bases upstream and downstream flanking the coding sequence.

## Results

### Patient's phenotype

Clinical findings of all 28 affected individuals enrolled in this study were shown in Supporting Information Table Is. The mean age of onset symptoms was 3 years. The mean Hb value was 10 g/dL before splenectomy, and mean ferritin value was 303 μg/L. Mean reticulocyte absolute count at diagnosis was 88797 × 10^6^/L. Clinical data shown here are indicative of a classical framework of CDA II; moreover, they are compatible with those published before [14]. For most of all patients SDS-PAGE revealed the typical narrower band size and faster migration of the band 3 and band 4.5 proteins; the diagnosis has been also confirmed by the demonstration of superficial appearance of reticulum-endothelial protein GRP78 on membrane proteins by WB.

### Identification of novel mutations of *SEC23B* gene in CDA II patients

**Figure 1:**
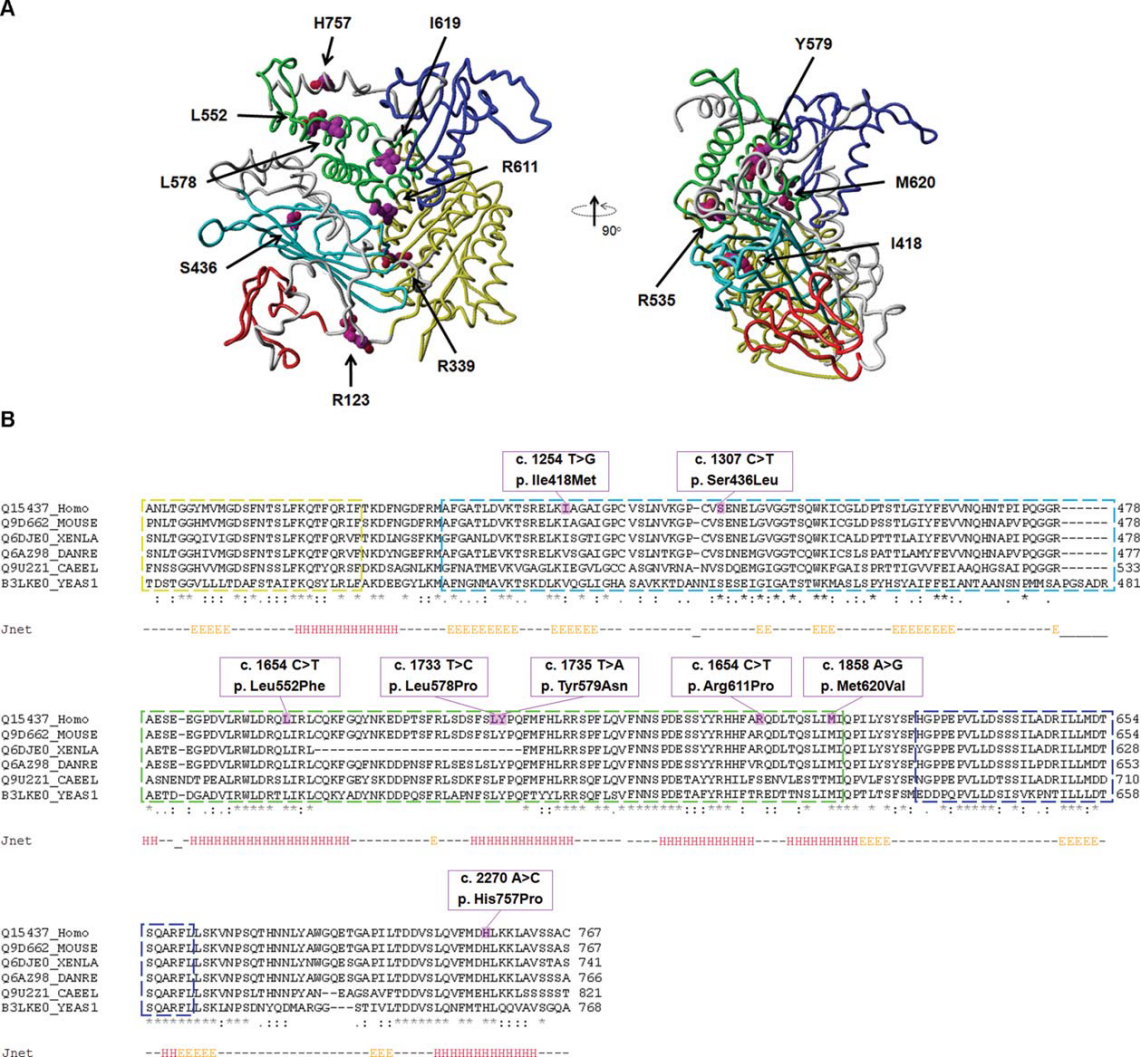
Localization and multiple-sequence alignment of mutations. Panel A. Localization of H757P, R611P, Y579N, I418M, M620V, L552F, S436L, L578P, R535X, R339X, and R123X mutations on 3D SEC23B protein structure predicted by ESyPred3D. From N-terminal to C-terminal: zinc finger (red), trunk (yellow), β-sheet (light blue), helical (green), and gelsolin-lile (blue) domains are shown. In grey are shown the inter-domains segments. Panel B. Six species were aligned using Database UniProtKB release 15.0 of Mar-25-2009; program NCBI BLASTP 2.0.12 (August 26, 2007) on http://services.uniprot.org (http://services.uniprot.org/blast/). At the bottom, a consensus line indicates conservation of the amino acid residues: an asterisk (*) indicates fully conserved sites, a colon (:) points to a conserved substitution, and a point (.) to a semi-conserved substitution. Protein secondary structure, predicted by Jnet neural network, is also shown: alpha helix (H), beta sheet (E), or random coil (-). Dotted lines define the domains of the protein SEC23B, according to the color scheme explained in panel A legend. Novel missense mutations are indicated with a red spot upon the sequence at their respective amino acid positions. β. [Color figure can be viewed in the online issue, which is available at wileyonlinelibrary.com.]

All enrolled patients were diagnosed at molecular level. Almost all patients carried two mutations in the compound heterozygous or homozygous state: only in four cases, mutations were not identified in both alleles. We identified a total of 19 novel different allelic variants (Table I) (Fig. 1A,B). Of these, eight were missense mutations: c.2270 A>C p.H757P, c.1832 G>C p.R611P, c.1858 A>G p.M620V; c.1735 T>A p.Y579N, c.1254 T>G p.I418M, c.1654 C>T p.L552F, c.1307 C>T p.S436L, c.1733 T>C p.L578P; 3 were nonsense mutations: c.1015 C>T p.R339X, c.1603 C>T p.R535X, c.367 C>T p.R123X; two were frameshift mutations: c.387 (del G) p.L129LfsX26, c.2150 (del C) p.A717VfsX7; 5 were splicing mutations: c.2149−2 A>G, c.1109+1 G>A, c.279+3 A>G, c.1109+5 G>A, c.221+31 A>G; only one consist of a deletion of a codon: c.1857_1859delCAT p.I619del. The 3D model of the protein has been built using the 3D structure 2NUP chain “A” (RCBS Protein Data Bank) as template. This template shares 84.5% identities with our query sequence (Fig. 1A).

**TABLE I:** Mutations Detected in *SEC23B*

Patient ID	Exon Intron	Nucleotide change^a^	Predicted effect on coding sequence^b^	Effect on protein or mRNA	Functional domains	References
F29P1	4	325 G>A	Glu109Lys	<5% of WT expression	Segment 2	[12]
	20	2270 A>C	His757Pro	Probably damaging (3.488)^c^ (−4.29/0.78)^d^	Segment 6	This study
F30P1	2	40 C>T	Arg14Trp	<5% of WT expression	Segment 1	[12]
	18–19	2149−2 A>G	–		Gelsolin	This study
F31P1	16	1858 A>G	Met620Val	Probably damaging (3.116)^c^ (−3.21/0.55)^d^	Helical	This study
	16	1858 A>G	Met620Val			
F32P1 F32P2	2	40 C>T	Arg14Trp	<5% of WT expression	Segment 1	[12]
	9–10	1109 + 1 G>A	–	Donor site abolition (0.93>–)^e^	Trunk	This study
F33P1	18–19	2149−2 A>G	–		Gelsolin	This study
F34P1 F34P2	2	40 C>T	Arg14Trp	<5% of WT expression	Segment 1	[12]
	5	387 (delG)	Leu129LeufsX26		Trunk	This study
F35P1 F35P2 F35P3	2	40 C>T	Arg14Trp	<5% of WT expression	Segment 1	[12]
	16	1832 G>C	Arg611Pro	Probably damaging (2.504)^c^ (−4.17/0.76)^d^	Helical	This study
F36P1	2	40 C>T	Arg14Trp	<5% of WT expression	Segment 1	[12]
	15	1735 T>A	Tyr579Asn	Probably damaging (2.992)^c^ (−4.64/0.84)^d^	Helical	This study
F37P1	11	1254T>G	Ile418Met	Probably damaging (1.851)^c^ (−3.87/0.71)^d^	β-sheet	This study
F38P1	9	1015 C>T	Arg339X		Trunk	This study
F39P1 F39P2	2	40 C>T	Arg14Trp	<5% of WT expression	Segment 1	[12]
	14	1603 C>T	Arg535X		Helical	This study
F40P1	14	1654 C>T	Leu552Phe	Probably damaging (2.125)^c^ (−4.12/0.75)^d^	Helical	This study
F41P1	4	325 G>A	Glu109Lys	<5% of WT expression	Segment 2	[12]
	11	1307 C>T	Ser436Leu	Probably damaging (2.577)^c^ (−3.09/0.52)^d^	β-sheet	This study
F42P1	3–4	279 + 3 A>G	–	Donor site alteration (1.00>0.95)^e^	Zink finger	This study
	4	325 G>A	Glu109Lys	<5% of WT expression	Segment 2	[12]
F43P1	13	1489 C>T	Arg497Cys		β-sheet	[12]
	14	1603 C>T	Arg535X		Helical	This study
F44P1	13	1489 C>T	Arg497Cys		β-sheet	[12]
	19	2150 (delC)	Ala717ValfsX7		Gelsolin	This study
F45P1 F45P2	2	40 C>T	Arg14Trp	<5% of WT expression	Segment 1	[12]
	9	1015 C>T	Arg339X		Trunk	This study
F46P1	15	1733 T>C	Leu578Pro	Probably damaging (2.370)^c^ (−4.06/0.74)^d^	Helical	This study
	15	1733 T>C	Leu578Pro			
F47P1	2	40 C>T	Arg14Trp	<5% of WT expression	Segment 1	[12]
	9–10	1109 + 5 G>A	–	Donor site abolition (0.93>–)^e^	Trunk	This study
F48P1	2–3	221 + 31 A>G	–	New donor site creation (–>0.99)^e^	Zink finger	This study
	5	367 C>T	Arg123X		Segment 2	
F49P1 F49P2	2	40 C>T	Arg14Trp	<5% of WT expression	Segment 1	[12]
	16	1857_1859delCAT	I619del		Helical	This study

a The nucelotides are numbered from the A of the ATG initiation codon (ENST00000377475).

b Accession number: Q15437 (UniProtKB/Swiss-Prot).

c PolyPhen prediction (PSIC score).

d PANTHER prediction (subPSEC score/*P*_deleterious_).

e Splice Site Prediction by Neural Network (WT sequence score > mutated sequence score).

Analysis of 120 control chromosomes showed that these changes were absent in normal subjects. Mutated positions were mostly conserved in analyzed species from *Homo* *sapiens* to yeast (Fig. 1B).

### Evaluation of the causal role of mutations by in silico analyses

We evaluated in silico the possible effect of amino acid substitutions of the eight missense mutations by the empirically derived rules of PolyPhen tool. The effect of these alterations on protein structure was predicted to be “probably damaging”, i.e., they are with high confidence supposed to affect protein function or structure (Table I). By using PANTHER tool, we partially confirmed the predicted functional significance of almost all missense mutations: only the M620V and the S436L variants are predicted to affect minimally protein function (subPSEC −3.2 and −3.09, respectively) (Table I).

Moreover, two (c.1832 G>C p.R611P, c.1654 C>T p.L552F) out of the eight missense mutations were predicted to alter the secondary structure of SEC23B mRNA (GeneBee program) when compared to that predicted for wild type sequence. Particularly, the free energy of SEC23B mRNA was predicted to be affected in a different manner by the two nucleotide substitution (−640.8 kcal/mol for wild type allele; −650.8 kcal/mol for c.1832 C allele; −636.5 kcal/mol for c.1654 T allele) (Supporting Information Fig. 1s, panels A-C-D). As positive control, we predicted also the secondary structure of c.325 A, the most frequent mutated allele so far described in CDA II patients, for which the free energy was predicted to be lower (−631.3 kcal/mol) when compared to wild type sequence (Supporting Information Fig. 1s, panel B). This observation is consistent with published observation: in fact, E109K is already associated with protein instability, with less than 5% of protein detectable compared to wild type [12].

Splice Site Prediction by Neural Network tool predicted a donor splice site abolition for two out the five splice site mutations, c.1109+1 G>A and c.1109+5 G>A, both falling in intron between exon 3 and 4. Instead, for the mutation c.221+31 A>G was predicted the creation of a new donor splice site with a high score (0.99). Finally, the mutation c.279+3 A>G seems to induce only a limited score reduction of the predicted wild type donor site (from 1.00 to 0.95) (Table I).

For only one splice site alteration, c.2149−2 A>G, this web server tool failed to predict the wild type acceptor splice site. For this variant, we used another bioinformatic tool, Gene Splicer (http://www.cbcb.umd.edu/software/GeneSplicer/gene_spl.shtml), whose prediction showed a mislocalization of the acceptor splice site (upstream of two positions) with a slight reduction of the score (from 6.5 to 6.3).

### Frequency of the mutations

**Figure 2:**
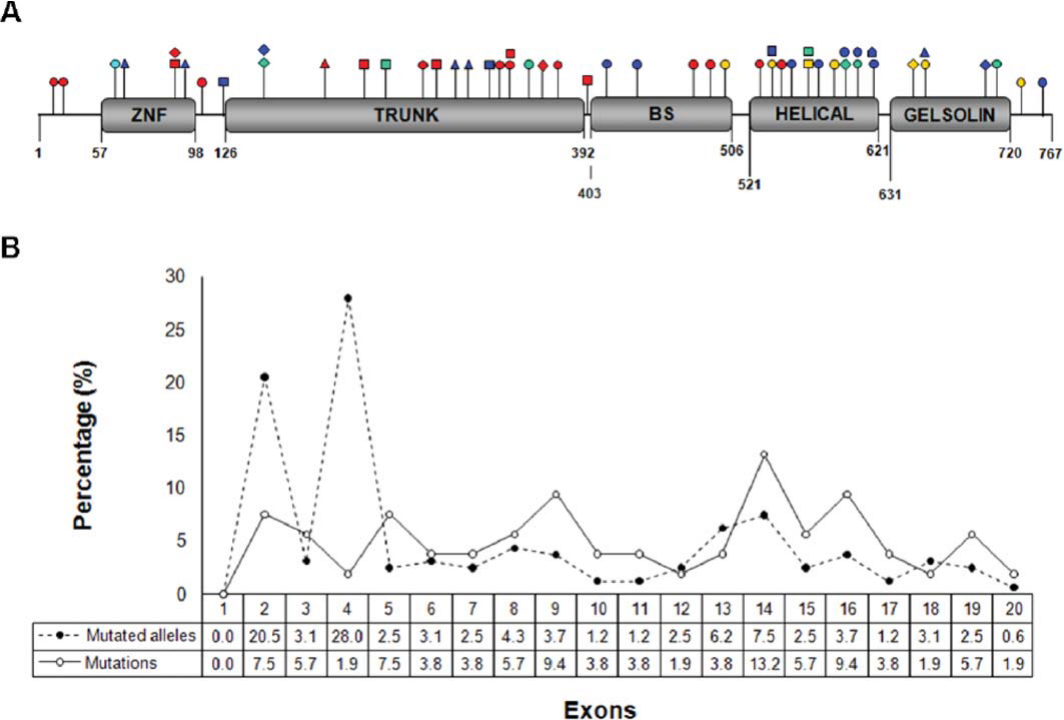
Exon allelic frequency and protein structure. Panel A. Localization of all novel mutations in bi-dimensional SEC23B protein structure. The domains are depicted in grey and for each domain the start and the end are indicated. In the upper part, mutations described until now are indicated with symbols and colors. The red color indicates the mutations first described by Schwarz et al., 2009; the green color indicates those first described by Bianchi et al., 2009; the yellow color indicates the novel variants described by Iolascon et al., 2009; the light blue color the novel mutation described by Fermo et al., 2010. The mutations described first in this study are shown in dark blue. ○ missense mutation; □ stop codon mutation; Δ splice site mutation, ⋄ frameshift mutation; amino acid deletion. Panel B. The relative richness of single mutations in each exon (solid line) has been assessed on a total count of 53 mutations. The frequency of mutated alleles for each exon (dotted line) has been calculated on an overall count of 161 mutated alleles. [Color figure can be viewed in the online issue, which is available at wileyonlinelibrary.com.]

To date, 34 causative mutations of CDA II in *SEC23B* gene were described [12–15], localized along the entire coding sequence of the gene. Here, we described 19 novel variants, bringing the total to 53 causative mutations (Fig. 2A).

Figure 2B shows the relative richness of (i) distinct mutations for each exon (solid line), as well as the frequency of (ii) mutated alleles for each exon (dotted line). On the basis of first analysis (i), the greatest number of different mutations falls in the exon 14 (13.2%), 9 and 16 (9.4%), 2 and 5 (7.5%). When we analysed the frequency of mutated alleles for each exon (ii), we found that the largest percentage ranks within the exons 2 (20.5%) and 4 (28.5%), as the most frequent mutations R14W, E109K found in CDA II patients occur within the exons 2 and 4, respectively.

## Discussion

CDA II is an autosomal recessive disorder affecting the normal differentiation-proliferation pathway of the erythroid lineage. Up to now, 4 articles described mutations in *SEC23B* gene as causative of CDA II [12–15]. Mutations previously identified in CDA II patients (Fig. 2A) are heterogeneous and include frameshift, splicing, deletion, missense, and nonsense variations. In this study, we describe 28 cases from 21 unrelated families with at least one affected proband, in which the diagnosis was made mainly on the basis of biochemical and cytological parameters. In almost all cases, *SEC23B* gene molecular analysis confirmed the diagnosis. We identified a total of 19 novel different allelic variants localized along the entire coding sequence (Fig. 2A, Table I). Most of the patients are compound heterozygotes for two different mutations, while only two are homozygotes (F31P1, F46P1). Patients F33P1, F37P1, F38P1, and F40P1, indeed, show only one mutation (2149−2 A>G, I418M, R339X, and L552F, respectively) in the heterozygous state. However, we neither analyzed the sequence of the promoter region nor did we look for the presence of any large DNA deletions, even if the presence of heterozygous state of several polymorphisms seems to exclude this possibility. As we already assumed, we hypothesized that the elusive mutation lies in *SEC23B*, because a compound heterozygosity is more likely than two simple heterozygosities, supposing that the second mutation stands at a different locus [14]. Our cohort of patients showed clinical parameters consistent with what we published before, insofar as patients carrying two missense mutations tend to be more mildly affected compared to those with the association of one nonsense and one missense mutations [14] (Supporting Information Table Is). Again, in no case did we find both alleles with a nonsense mutation. This confirm that the absence of SEC23B is supposed to be lethal. The pattern of these mutations emphasizes the high allelic heterogeneity of this condition, as most of the variations are inherited as private mutations. However, we also confirmed that in CDA II there are recurring alleles, as already demonstrated [14].

This study provided a brief description of a novel set of *SEC23B* mutations in CDA II disease, localized along the entire coding sequence and divided into: eight missense, two frameshift, three nonsense, five splicing mutations, and one amino acid deletion. The frameshift and nonsense mutations result in the formation of a shorter protein with the loss of some domains. Particularly, the nonsense c.1015 C>T p.R339X and the frameshift c.387 (delG) p.L129LfsX26 cause the loss of part of the trunk domain, fundamental for the interaction with the partners of the “pre-budding complex,” Sec24 and Sar1. The causative role of the missense changes was inferred first by the highly evolutionary conservation of the replaced residues (Fig. 1B) and then by in silico analysis. We attempted to characterize the putative effects of the amino acid substitutions on protein structure or function by using PolyPhen tool. The empirically derived rules of this web server tool predicted for all missense mutations a “probably damaging” effect on protein structure, i.e., they are with high confidence supposed to affect protein function or structure (Table I). Of note, the c.2270 A>C p.H757P substitution, which showed the highest PSIC score (3.488) by PolyPhen, is the first mutation identified to date that fall in the exon 20, only 10 amino acids prior to the end of the protein. Also PANTHER confirmed the probability that this variant might have a deleterious effect on protein function, with a *P*_deleterious_ = 0.78 (subPSEC = −4.2). Since it has been already demonstrated that the most frequent mutation, c.325 G>A (p.E109K), lead to a protein instability [12], we decided to analyze the effect of all eight missense substitution on mRNA stability. For this purpose, we used a freeware software program, GeneBee, in which we loaded as the input file the full-length sequence of SEC23B mRNA, either wild type or mutated alleles. The server predicted that only two (c.1654 C>T p.L552F, c.1832 G>C p.R611P) out of the eight missense mutations might to alter the secondary structure of SEC23B mRNA when compared to that predicted for wild type sequence. Particularly, for the c.1654 C>T (p.L552F), the free energy of SEC23B mRNA was predicted to be higher (−636.5 kcal/mol) when compared to that of wild type (−640.8 kcal/mol); this value is suggestive of a reduced mRNA stability, which could result in reduced expression of SEC23B mRNA (Supporting Information Fig. 1s, panels A–C). Instead, the substitution c.1832 G>C (p.R611P) seems to confer a higher stability to mRNA secondary structure, with a predicted free energy of −650.8 kcal/mol (Supporting Information Fig. 1s, panel D). In the latter case, this variant could lead to the stabilization of mRNA resulting in increased expression of mutated allele. Of note, the free energy of our positive control, c.325 G>A (p.E109K), was predicted to be lower (−631.3 kcal/mol) when compared to wild type sequence (Supporting Information Fig. 1s, panel B). Since we did not have the availability of the cDNA of patients with mutations close to splicing sites, we attempted to define the effect of mutations near splice site on mRNA processing alteration by using the web server tool, Splice Site Prediction by Neural Network. For both variants, c.1109+1 G>A and c.1109+5 G>A, the donor splice site abolition has been predicted. Instead, for the mutation c.221+31 A>G was predicted the creation of a new donor splice site with a high score (0.99).

To provide a useful information for molecular diagnosis of CDA II, we assessed the relative richness of any distinct mutations in each exon of *SEC23B* gene, as well as the frequency of mutated alleles for each exon, including also the bases flanking the coding sequence. As most of the mutations are the results of sporadic and independent events, we analysed the relative richness of the single mutations for each exon, to contribute to the improvement of the patient management, of the molecular screening and for providing a more aware genetic counselling. On the basis of this analysis, the most richly mutated exon is the 14 (13.2%), followed closely by exons 9 and 16 (9.4%). Of note, the exon 4, from which the most frequently mutated alleles in CDA II patients (E109K) stem, showed a relative richness of mutations of only 1.9%. However, when we analysed the frequency of mutated alleles for each exon, we found that the most frequently mutated exon 4 (28.5%), closely followed by the exon 2 (20.5%), according to the most frequent mutations E109K and R14W (exons 4 and 2, respectively). Both these available information could facilitate the molecular approach in terms of exon priorities to be analyzed.

Altogether, our results extend the pattern of *SEC23B* mutations associated with CDA II, propose putative models of causality for almost all the 19 novel variants identified and, finally, provide powerful information to improve the molecular diagnosis of CDA II.
