# Supplementary material for: Mutational spectrum in congenital dyserythropoietic anemia type II: Identification of 19 novel variants in SEC23B gene
Source: Am J Hematol. 2010 Dec;85(12):915–20. doi: 10.1002/ajh.21866 (PMC3015065; doi:10.1002/ajh.21866)
Supplement: Supplementary file 1 [file ajh0085-0915-SD1.doc]

| **Table Is. Clinical data** | | | | | | | | |
| --- | --- | --- | --- | --- | --- | --- | --- | --- |
| **Patient ID** | **Gender** (m)ale or (f)emale | **Date of birth** | **Onset Symptoms** (years) | **Transfusions** (y)es or (n)ot | **Hb** g/dl | **Retics absolute count** | **Ferritin** µg/L | **Jaundice**  (y)es or (n)ot |
|
| F29P1 | m | 2004 | at birth | y | 8.7 | 106000 | 200 | y |
| F30P1 | m | 1988 | 3 | n | 12.2 | 40400 | 350 | y |
| F31P1 | f | 1973 | 0.2 | n | 11.5 | 49000 | 180 | y |
| F32P1 | f | 1971 | 6 | n | 9.0 | n.d. | 190 | n |
| F32P2 | f | 1963 | 2 | y | 8.0 | n.d. | 400 | n |
| F33P1 | m | 1995 | 2 | n | 9.5 | 98100 | 250 | n |
| F34P1 | f | 1987 | 0.8 | y | 9.5 | 88000 | 250 | n |
| F34P2 | f | 1987 | 0.8 | y | 9.2 | 58000 | 125 | y |
| F35P1 | f | 1991 | 3 | n.d. | 10.2 | 179000 | 213 | y |
| F35P2 | m | 1993 | 0.3 | y | 8.6 | 185000 | 242 | y |
| F35P3 | f | 1995 | 0.6 | y | 10.1 | 135000 | 25 | y |
| F36P1 | m | 1990 | 3 | y | 10.2 | 112460 | 333 | y |
| F37P1 | m | 2008 | at birth | y | 7.4 | 115000 | 45 | y |
| F38P1 | m | 1990 | n.d. | n.d. | 8.9 | 73510 | 280 | y |
| F39P1 | f | 1999 | 7 | n.d. | 9.3 | 87000 | 187 | y |
| F39P2 | f | 1999 | 5 | n.d. | 9.5 | 33000 | 206 | y |
| F40P1 | m | 1966 | 19 | n | 10.6 | 90000 | 487 | y |
| F41P1 | m | 1989 | n.d. | n | 10.2 | 98000 | 212 | y |
| F42P1 | f | 1985 | n.d. | n.d. | 9.3 | 82689 | 659 | y |
| F43P1 | f | 1992 | n.d. | y | 9.1 | 83104 | 750 | y |
| F44P1 | m | 1996 | n.d. | y | 8.8 | 68000 | 712 | y |
| F45P1 | m | 1987 | 3 | y | 8.7 | 67260 | 600 | n |
| F45P2 | m | 1991 | 2 | y | 9.6 | 27900 | 430 | y |
| F46P1 | f | 1998 | 0.6 | y | 8.0 | n.d. | 196 | y |
| F47P1 | m | 1999 | 2.2 | y | 6.5 | 52000 | n.d. | n |
| F48P1 | m | 1989 | 2.6 | n | 10.4 | 136510 | 55 | y |
| F49P1 | m | 1999 | at birth | n | 12.8 | 80000 | 212 | y |
| F49P2 | f | 1999 | at birth | n | 10.9 | 75000 | 382 | y |
| n.d. not determined | | | |  |  |  |  |  |

**Figure 1s. mRNA Secondary Structure prediction**

Panel A. Secondary structure of the wild type sequence of SEC23B mRNA full length predicted by GeneBee program. The free energy value has been predicted -640.8 kcal/mol.

Panel B. Secondary structure of the mutated sequence (c.325 G>A, p.E109K) of SEC23B mRNA full length predicted by GeneBee program. The free energy value has been predicted -631.3 kcal/mol for c.325 C allele.

Panel C. Secondary structure of the mutated sequence ( c.1654 C>T, p.L552F) of SEC23B mRNA full length predicted by GeneBee program. The free energy value has been predicted -636.5 kcal/mol for c.1654 T allele.

Panel D. Secondary structure of the mutated sequence (c.1832 G>C, p.R611P) of SEC23B mRNA full length predicted by GeneBee program. The free energy value has been predicted -650.8 kcal/mol for c.1832 C allele.


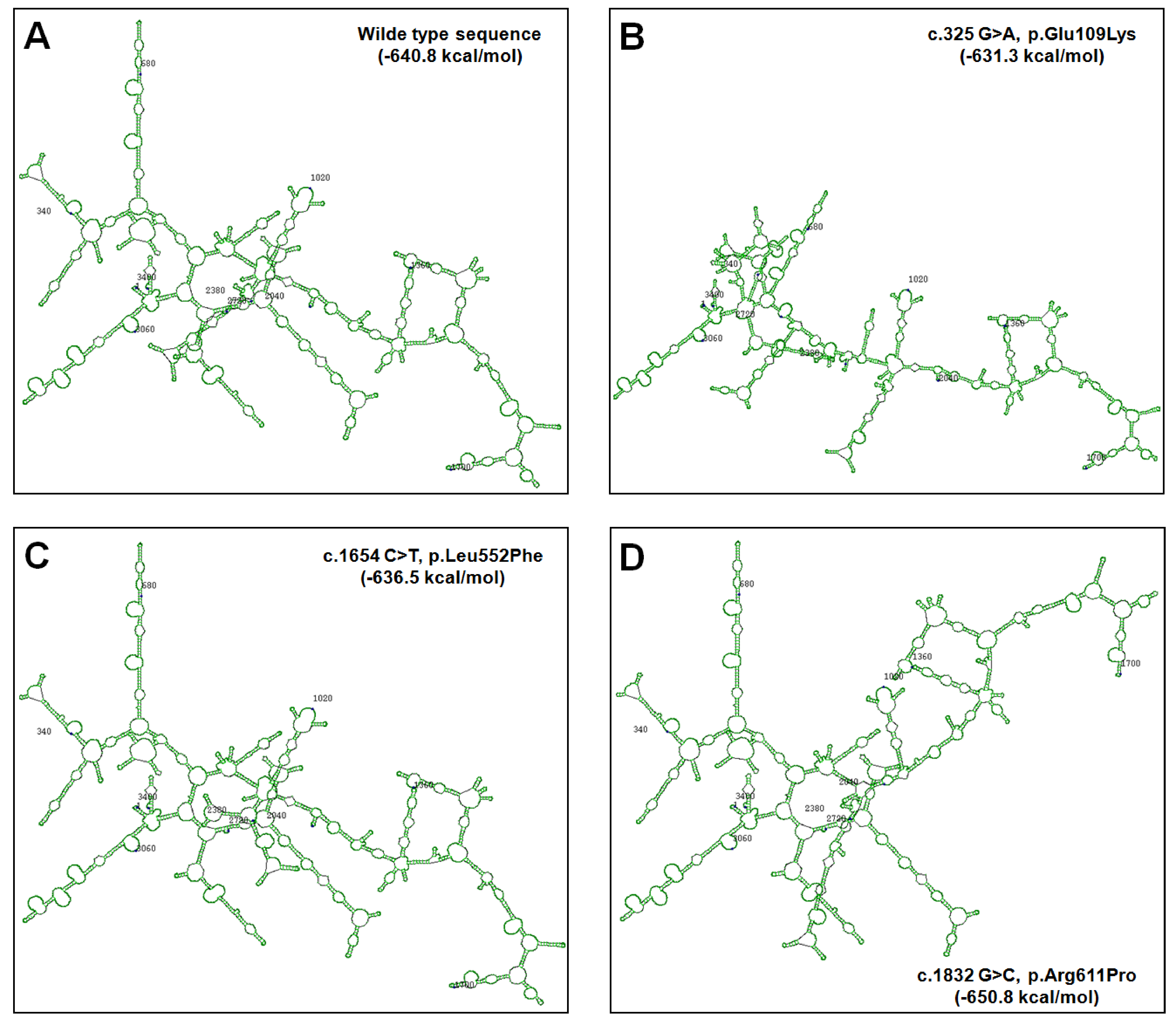


**Figure 1s**
